# Slow Acceptance of Universal Antiretroviral Therapy (ART) Among Mothers Enrolled in IMPAACT PROMISE Studies Across the Globe

**DOI:** 10.1007/s10461-019-02624-3

**Published:** 2019-08-09

**Authors:** L. Stranix-Chibanda, S. Brummel, J. Pilotto, M. Mutambanengwe, V. Chanaiwa, T. Mhembere, M. Kamateeka, J. Aizire, G. Masheto, R. Chamanga, M. Maluwa, S. Hanley, E. Joao, G. Theron, N. Nevrekar, M. Nyati, B. Santos, L. Aurpibul, M. Mubiana-Mbewe, R. Oliveira, T. Anekthananon, P. Mlay, K. Angelidou, C. Tierney, L. Ziemba, A. Coletti, K. McCarthy, M. Basar, N. Chakhtoura, R. Browning, J. Currier, M. G. Fowler, P. Flynn, for the PROMISE study team

**Affiliations:** 1 University of Zimbabwe College of Health Sciences, Paediatrics and Child Health, Harare, Zimbabwe; 2 Harvard T.H. Chan School of Public Health, Center for Biostatistics in AIDS Research in the Department of Biostatistics, Boston, USA; 3 Laboratorio de AIDS e Imunologia Molecular - Fiocruz, Hospital Geral de Nova Iguacu, Rio de Janeiro, Brazil; 4 University of Zimbabwe College of Health Sciences – Clinical Trials Research Centre, 15 Phillips Avenue, Belgravia, Harare, Zimbabwe; 5 Makerere University - Johns Hopkins University Research Collaboration, Kampala, Uganda; 6 Harvard T.H. Chan School of Public Health, Botswana Harvard AIDS Institute Partnership, Gaborone, Botswana; 7 College of Medicine - Johns Hopkins Research Project, Blantyre, Malawi; 8 University of North Carolina Project, Lilongwe, Malawi; 9 Centre Aids Prevention Research South Africa (CAPRISA), University of KwaZulu-Natal, Durban, South Africa; 10 Department of Infectious Diseases, Hospital Federal dos Servidores do Estado, Rio de Janeiro, Brazil; 11 Department of Obstetrics and Gynaecology, Stellenbosch University, Cape Town, South Africa; 12 Department of Obstetrics and Gynaecology, BJ Government Medical College, Pune, India; 13 Perinatal HIV Research Unit, Johannesburg, South Africa; 14 Hospital Nossa Senhora da Conceicao, Porto Alegre, Brazil; 15 Research Institute for Health Sciences, Chiang Mai University, Chiang Mai, China; 16 Centre for Infectious Disease Research in Zambia, Lusaka, Zambia; 17 Instituto of Pediatrics Federal University of Rio de Janeiro, Rio de Janeiro, Brazil; 18 Faculty of Medicine Siriraj Hospital, Mahidol University, Bangkok, Thailand; 19 Kilimanjaro Christian Medical Centre, Moshi, Tanzania; 20 FHI 360, IMPAACT Operations Center, Durham, NC USA; 21 Frontier Science and Technology Research Foundation, Amherst, USA; 22 Eunice Kennedy Shriver National Institute of Child Health and Human Development, Bethesda, USA; 23 Division of AIDS, National Institute of Allergy and Infectious Diseases, Bethesda, USA; 24 Division of Infectious Diseases, University of California Los Angeles, Los Angeles, USA; 25 Department of Pathology, Johns Hopkins University School of Medicine, Baltimore, USA; 26 Department of Infectious Diseases, St Jude Children’s Research Hospital, Memphis, TN USA

**Keywords:** Treat All, Universal ART, Women with HIV

## Abstract

The PROMISE trial enrolled asymptomatic HIV-infected pregnant and postpartum women not eligible for antiretroviral treatment (ART) per local guidelines and randomly assigned proven antiretroviral strategies to assess relative efficacy for perinatal prevention plus maternal/infant safety and maternal health. The START study subsequently demonstrated clear benefit in initiating ART regardless of CD4 count. Active PROMISE participants were informed of results and women not receiving ART were strongly recommended to immediately initiate treatment to optimize their own health. We recorded their decision and the primary reason given for accepting or rejecting the universal ART offer after receiving the START information. One-third of participants did not initiate ART after the initial session, wanting more time to consider. Six sessions were required to attain 95% uptake. The slow uptake of universal ART highlights the need to prepare individuals and sensitize communities regarding the personal and population benefits of the “Treat All” strategy.

## Introduction

### Previous Antiretroviral Strategies for Pregnant Women

Prior to 2016, ART was reserved for HIV-infected pregnant women with signs of immunosuppression or clinical AIDS [1]. Women who were not immunocompromised received antiretroviral prophylaxis consisting of mono-, dual or triple antiretroviral regimens throughout pregnancy, and they or their infants received prophylaxis during lactation. Significant resources were invested in educating communities about the immunological threshold for ART initiation and increasing access to CD4 cell testing in maternity clinics. The PROMISE study was a strategy trial designed to compare these antiretroviral strategies among asymptomatic HIV-infected pregnant women who did not meet country criteria for ART initiation, assessing vertical HIV transmission during pregnancy and post-delivery, infant safety and maternal health.

### Universal ART Approach

The current World Health Organization (WHO) recommendation for women diagnosed with HIV infection in pregnancy is to initiate life-long triple ART regardless of clinical or immunological staging [2]. This “Treat All” approach was informed in 2015 by the START trial [3] which demonstrated that universal ART initiation reduces the risk of HIV disease progression. Previously, concerns had been voiced about the acceptability of this approach, given the prevailing perception in communities that ART was reserved for people who were sick with low CD4 counts based on prior guidance [1, 2]. Similarly, the practicality of delivering such a strategy on a public scale and the ability of women to adhere to satisfactory levels had been questioned because of multiple operational challenges [4–7].

### PROMISE Response to START Study Results

Upon release of the START study results in July 2015, the PROMISE randomized interventions were immediately halted, and universal ART was recommended by the study team to all participants. Since the uptake of life-long ART would be relevant to ART programs that would soon incorporate this change, quantitative data were collected in a systematic manner from these asymptomatic women with high CD4 counts enrolled in a clinical trial across diverse global settings. We assessed the uptake of universal ART and present reasons to either accept or decline the recommendation.

## Methods

### PROMISE Study Design

PROMISE was conducted at 70 research sites in 15 countries within sub-Saharan Africa, Asia and the Americas. A total of 5400 asymptomatic HIV-infected pregnant women with high CD4 counts (above 350 cells/mm^3^ or the treatment threshold at that time) were assigned to different ARV strategies and followed for HIV disease progression, vertical transmission and safety. In settings where maternal ART and replacement feeding was standard, eligible women were randomized within 6 weeks of delivery to continue or stop ART and remain in follow-up for intense monitoring of HIV disease progression and adverse events in a protocol named 1077HS (for HAART standard) [8]. In settings where maternal ART was not standard for the prevention of vertical transmission, separate protocols were conducted—1077FF and 1077BF—in formula feeding and breastfeeding settings, respectively. Within 1077FF/BF, pregnant women were randomized to triple ART or prophylaxis with zidovudine throughout pregnancy and delivery in the Antepartum Component plus single dose nevirapine at delivery followed by a 2 week “tail” of tenofovir/emtricitabine [9]. Women who did not access HIV services in pregnancy could join the study around the time of delivery. Eligible mothers were randomized after delivery in the Postpartum Component to receive or not receive maternal ART. Once the period of risk for vertical transmission was over—at delivery for 1077FF and after weaning or after 18 months of study intervention, whichever came first, for 1077BF—women receiving ART were randomized in the Maternal Health Component to continue or stop ART. Enrolled women who were not eligible for subsequent randomizations were followed in an observational cohort through study completion.

Participants were followed at least quarterly to monitor clinical, immunological and virologic status. Women randomly assigned to not take ART started ART once country criteria for treatment initiation were met. Women remained in PROMISE follow-up regardless of ART status.

### Participant Tracing and Information-Giving About the START Study Results

**Fig. 1:**
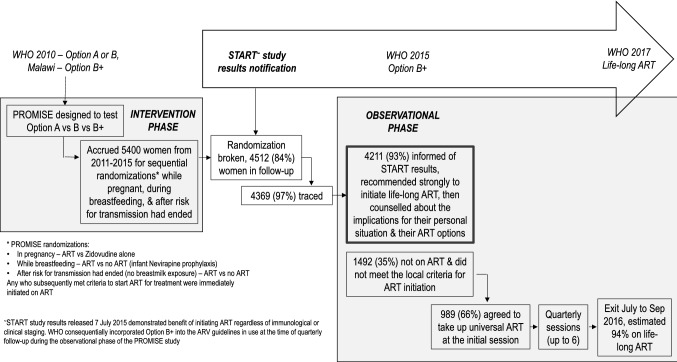
Timeline of events in the PROMISE study and concurrent ARV guidelines

Figure 1 illustrates the timeline of events surrounding the action taken in response to the release of the START study results. The PROMISE study team directed sites to actively contact participants to return to the clinic to receive important information that could influence their decision to remain in follow-up. The rate of return was tracked at each site to ensure a timely response. The study team provided a structured script of simple talking points developed with input from the community and sites, although not formally piloted. After covering the essential elements of the START study population and findings, the script included a recommendation that all women in PROMISE take ART, seeing the START study had showed that it is better to start ART before a decline in CD4 count.

When the participants returned to the study clinic, trained staff revisited the content of the PROMISE informed consent discussion with the women, emphasizing the study rationale, randomizations and role of the CD4 count in determining eligibility for initiating ART for the purposes of maternal treatment. The START study talking points were then delivered in a language chosen by the participant. Women already receiving ART were told that they would remain on ART and in follow-up through scheduled study exit in September 2016. Women not on ART were strongly advised to accept the offer to immediately initiate ART as the best antiretroviral strategy to preserve their own health. Participants were informed that remaining in follow-up was not dependent on being on ART; their willingness to continue participation was their choice. A discussion followed to verify comprehension of the implications for the individual study participant and address any questions raised.

### Individual Counselling

The comprehension discussion lead into an individual counselling session which followed the site’s routine counselling procedures. The approach adopted by the numerous counsellors across the different sites was not standardized due to the rapid nature of the action taken, and sessions were not audio-recorded. Women were encouraged to give their personal interpretation of the START results and to explore their reaction to the strong recommendation for all women with HIV to take ART in a free-ranging discussion. They could take as much time as they needed in the session to work out their feelings about continued study participation and, if relevant, to decide whether they wanted to accept or decline the offer of universal ART. For participants advised to initiate universal ART because of the START results, counsellors probed each woman to give her reasons for accepting or rejecting the offer, then to select her primary reason for doing so.

The study physician subsequently completed the routine study review and linkage with appropriate treatment services for those initiating ART. Participants who chose not to initiate universal ART at the initial session repeated the process at subsequent visits scheduled quarterly until ART initiation or through study exit.

### Data Collection and Coding

After the counselling sessions, counsellors completed a structured data form to capture the information delivery process and decisions made. They categorized the responses into pre-set closed options derived from study team discussions with the community representatives and sites. They recorded additional detail as open text comments. Sites submitted data forms to a centralized database for analysis. These data forms were also completed at subsequent sessions for those participants who initially declined universal ART. Open text comments were subsequently categorized by two researchers following a code book developed by consensus through discussion of themes emerging from the line list of comments.

### Statistical Methods

These data analyses were based on women in follow-up off ART at the time of this action and with at least one counselling session. Frequencies, means, and percentiles were used for descriptive purposes. T-tests compared the mean difference between groups. The cumulative probability of remaining off ART over time was estimated using the Kaplan–Meier method. Sensitivity analyses were performed by using the antiretroviral drug record in place of the counselling form, with the conclusion that the analysis is robust to missing counselling session forms (data not shown). Analyses were performed using SAS version 9.4. P-values less than 0.05 were considered to be statistically significant.

Based on these data generated from a large, multi-site clinical trial population with prior access to intense HIV disease monitoring and extensive counselling services, we report the uptake of universal ART over time by women not receiving ART at the time the START study results were communicated. We describe the primary reasons women gave in support of the decisions they made.

## Results

### Accrual

At the start of the information and counselling sessions, 4512 of the 5400 PROMISE participants were still enrolled (84%) and the remainder had been lost to follow-up. All of the PROMISE women had delivered the index pregnancy. Of these, 4211 (93%) underwent at least one standardized counselling session. At the time of the initial session, the average duration of follow-up in PROMISE was 2.8 years (range 1–6 years), which reflects the minimum time they had knowledge of their HIV status. Two thousand seven hundred and nineteen women were on ART (65%). The remaining 1492 women (35%) were not on ART, as they did not meet the local criteria at that time and had not been randomized to an ART study arm.

### Baseline Characteristics and Health Status at Initial Session

Eighty-five percent of those who were not on ART and offered universal ART were Black or African American and 14% identified as Hispanic or Latina. Participants were from Sub-Saharan Africa (77%), South America (13%), Asia (7%) and North America (3%). The average age was 27.3 years, 92% were in WHO Clinical Stage I, the mean [95% confidence interval (CI)] CD4 count was 680.4 (667.6, 693.2) cells/mm^3^, and 26% had a HIV viral load below or equal to 1000 copies/mL (Table 1).

**Table 1:** Characteristics of women not on ART at first counselling session by PROMISE protocol

	PROMISE protocol		Total (N = 1492)
	1077BF/FF^a^ (N = 1036)	1077HS^b^ (N = 456)	
Age in years			
Mean (95% CI)	27.1 (26.8, 27.4)	27.9 (27.4, 28.5)	27.3 (27.1, 27.6)
Median	26.6	27.8	26.9
10%, 90%	20.5, 34.3	20.5, 35.6	20.5, 34.8
Ethnicity			
Not Hispanic or Latina	993 (96%)	254 (56%)	1,247 (84%)
Hispanic or Latina	6 (1%)	197 (43%)	203 (14%)
Patient does not know	37 (4%)	4 (1%)	41 (3%)
Ethnicity unavailable to clinic	0 (0%)	1 (0%)	1 (0%)
Race			
Black or African American	999 (96%)	267 (59%)	1,266 (85%)
Asian	37 (4%)	71 (16%)	108 (7%)
White	0 (0%)	74 (16%)	74 (5%)
Other	0 (0%)	31 (7%)	31 (2%)
Unknown	0 (0%)	9 (2%)	9 (1%)
More than one race	0 (0%)	3 (1%)	3 (0%)
American Indian	0 (0%)	1 (0%)	1 (0%)
CD4 Count (cells/mm^3^)			
Mean (95% CI)	708.8 (694.1, 723.6)	615.6 (591.6, 639.6)	680.4 (667.6, 693.2)
Median	665	545.5	637
10%, 90%	455, 1,054	364, 971	406, 1,042
≥ 500	851 (82%)	278 (61%)	1,129 (76%)
350–499	157 (15%)	141 (31%)	298 (20%)
< 350	27 (3%)	35 (8%)	62 (4%)
WHO clinical stage			
Clinical stage I	941 (91%)	427 (94%)	1,368 (92%)
Clinical stage II	81 (8%)	22 (5%)	103 (7%)
Clinical stage III	10 (1%)	5 (1%)	15 (1%)
Clinical stage IV	1 (0%)	0 (0%)	1 (0%)
Log viral load (copies/mL)			
Mean (95% CI)	3.6 (3.5, 3.6)	3.6 (3.5, 3.7)	3.6 (3.5, 3.6)
Median	3.7	3.8	3.7
10%, 90%	2.2, 4.8	2.0, 4.6	2.1, 4.7
Viral load (copies/mL)			
> 200	820 (87%)	368 (87%)	1,188 (87%)
≤ 200	118 (13%)	56 (13%)	174 (13%)
Viral load (copies/mL)			
> 1000	694 (74%)	316 (75%)	1,010 (74%)
≤ 1000	244 (26%)	108 (25%)	352 (26%)

One participant in WHO clinical stage IV was not on ART due to missed study visits. This woman started ART once the stage IV was identified

^a^1077BF/FF: breastfeeding and formula feeding versions of the PROMISE protocol

^b^1077HS: HAART standard version of the PROMISE protocol

### Universal ART Uptake

**Fig. 2:**
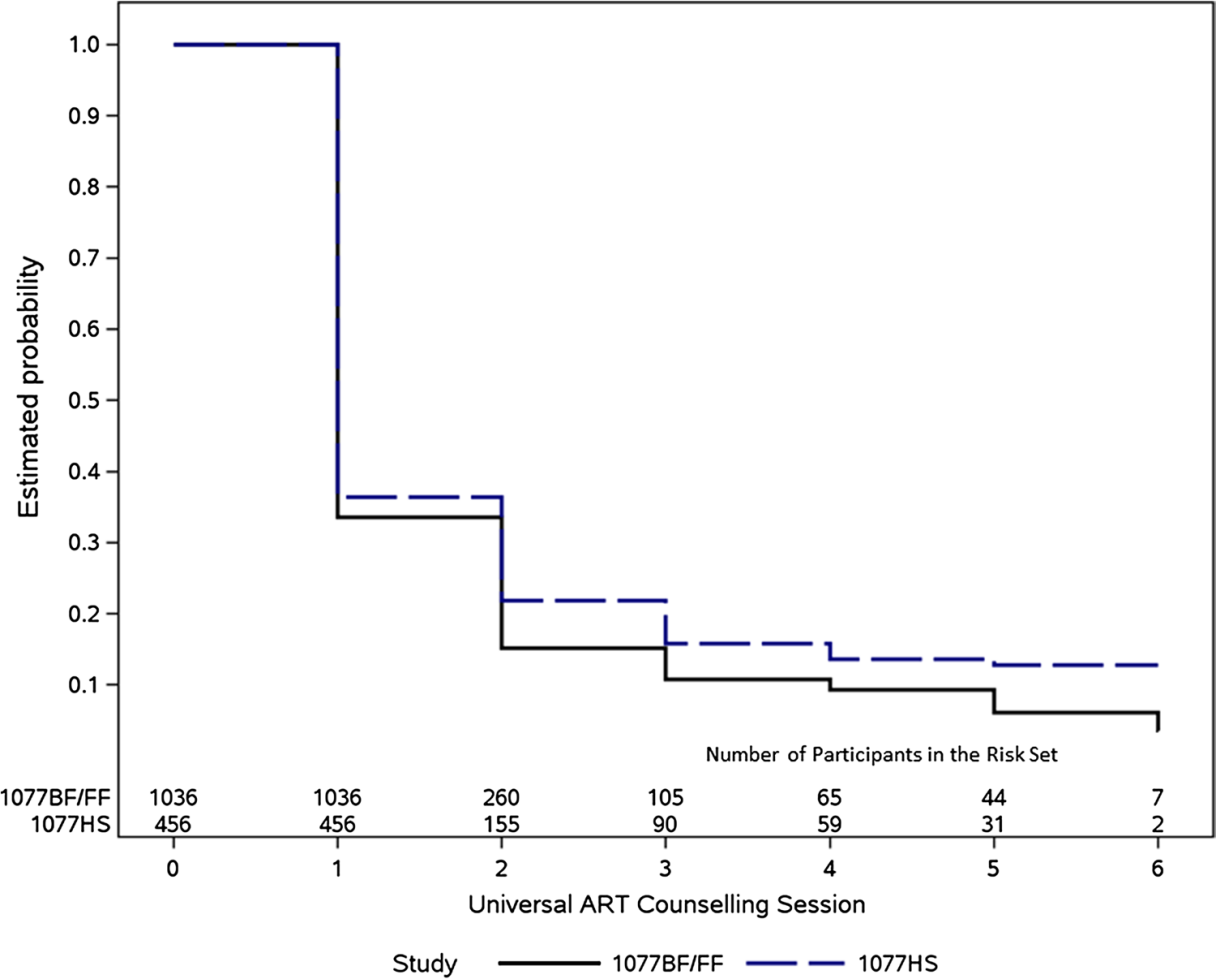
Kaplan–Meier estimate of the probability of remaining off ART as a function of the number of times counseled

With one counselling session, the Kaplan–Meier estimate of the percentage that started universal ART was 65.5% (95%CI 63%, 68%). The average days between subsequent sessions was 79 days and the maximum number of sessions was six. The probability of being on universal ART increased after each subsequent session (Fig. 2): 82.6% after two counselling sessions, 87.5% after three sessions and 94.4% at study end 1 year later. Initial ART uptake varied widely by country (Table 2).

**Table 2:** Kaplan–Meier estimates of the probability to subsequent uptake of universal ART by country

PROMISE protocol	Country	Counselling session					
		1	2	3	4	5	6
Overall	Overall	65.5% (63%, 68%)	82.6% (80%, 85%)	87.5% (86%, 89%)	89.2% (87%, 91%)	91.6% (90%, 93%)	94.4% (91%, 97%)
1077HS^b^	Argentina	85.7% (63%, 98%)	85.7% (63%, 98%)	92.9% (72%, 100%)			
	Botswana	61.8% (54%, 70%)	79.1% (72%, 85%)	90.3% (85%, 95%)	92.3% (87%, 96%)	92.3% (87%, 96%)	
	Brazil	64.6% (58%, 72%)	80.3% (74%, 86%)	83.7% (78%, 89%)	85.6% (80%, 90%)	87.8% (82%, 92%)	
	Haiti	92.9% (72%, 100%)	92.9% (72%, 100%)	92.9% (72%, 100%)	92.9% (72%, 100%)	92.9% (72%, 100%)	
	Thailand	50.7% (40%, 63%)	66.7% (56%, 77%)	72.7% (62%, 83%)	74.3% (64%, 84%)	74.3% (64%, 84%)	74.3% (64%, 84%)
	USA	66.7% (48%, 84%)	72.2% (53%, 88%)	72.2% (53%, 88%)	79.2% (59%, 94%)	79.2% (59%, 94%)	
1077BFFF^a^	India	83.8% (70%, 93%)	89.2% (77%, 97%)	91.9% (80%, 98%)	91.9% (80%, 98%)	91.9% (80%, 98%)	91.9% (80%, 98%)
	Malawi	81.4% (76%, 86%)	91.8% (88%, 95%)	93% (89%, 96%)	93.8% (90%, 97%)	94.7% (91%, 97%)	94.7% (91%, 97%)
	South Africa	48.5% (43%, 54%)	79.9% (74%, 85%)	85.2% (80%, 90%)	89.1% (84%, 93%)	93.8% (89%, 97%)	
	Tanzania	36.7% (22%, 56%)	46.7% (31%, 66%)	57.3% (41%, 75%)	57.3% (41%, 75%)	63.4% (45%, 81%)	
	Uganda	55.6% (48%, 63%)	86% (80%, 91%)	94.5% (90%, 97%)	95.1% (91%, 98%)	97.9% (95%, 99%)	
	Zambia	85.7% (68%, 96%)	85.7% (68%, 96%)	85.7% (68%, 96%)	85.7% (68%, 96%)		
	Zimbabwe	85.5% (80%, 90%)	89.7% (85%, 94%)	90.5% (86%, 94%)	91.3% (86%, 95%)	95.7% (90%, 99%)	

Probability of ART uptake (95% confidence interval)

Peru was omitted from this table because the sample size was not large enough to calculate ART uptake probability estimates

^a^1077BF/FF: breastfeeding and formula feeding versions of the PROMISE protocol

^b^1077HS: HAART standard version of the PROMISE protocol

### Primary Reasons Given

The primary reasons given for declining universal ART at the initial counselling session were wanting more time to consider (44%) or feeling well and knowing their CD4 count was high (19%). A minority expressed concern about potential side effects of ART (8%), that taking ART would lead to inadvertent disclosure of HIV status (7%) and about committing to life-long treatment (7%). Specific barriers were cited in the open text comments for 95 of the 201 participants with more detail recorded about why they wanted more time to consider the offer. The most frequently mentioned were the need to first consult significant others (32, 34%), psychological unpreparedness to make this important decision (25, 26%) and being preoccupied with a social commitment (6, 6%).

The primary reasons given for accepting universal ART at the initial counselling session were concern about health (46%), because of the recommendation given by the protocol team (36%), and concern about the CD4 count (16%) (Table 3). Women who selected concern about CD4 count as their primary reason for initiating universal ART had lower mean CD4 counts than women who stated that they felt well and knew their CD4 count was high; 617 cells/mm^3^ versus 878 cells/mm^3^ (mean difference = − 251, t-value = − 4.96, p < 0.001) in 1077BF/FF and 479 cells/mm^3^ versus 686 cells/mm^3^ (mean difference = 207, t-value = − 4.50, p < 0.001) in 1077HS.

**Table 3:** Primary reasons given to decline or accept the offer of universal ART at the initial counselling session

	1077BF/FF		1077HS		Total	
	N	(%)	N	(%)	N	(%)
Primary reason to decline						
Wants more time to consider	155	(46%)	63	(38%)	218	(44%)
Feels well/knows CD4 count is high	47	(14%)	48	(29%)	95	(19%)
Concerned about potential side effects	27	(8%)	13	(8%)	40	(8%)
Concerned about commitment to life-long ART	29	(9%)	6	(4%)	35	(7%)
Concerned about HIV disclosure	30	(9%)	5	(3%)	35	(7%)
Too busy with child care or other responsibilities	11	(3%)	9	(5%)	20	(4%)
Other reason	11	(3%)	8	(5%)	19	(4%)
Concerned about adherence	7	(2%)	12	(7%)	19	(4%)
Knows treatment not indicated per current local standard guidelines	18	(5%)	0	(0%)	18	(4%)
Total declined	335	(100%)	164	(100%)	499	(100%)
Primary reason to accept						
Concerned about health	321	(46%)	128	(44%)	449	(46%)
Understands that treatment is now recommended by the PROMISE study team based on the START results	244	(35%)	114	(39%)	358	(36%)
Concerned about CD4 count	112	(16%)	41	(14%)	153	(16%)
Other reason	16	(2%)	8	(3%)	24	(2%)
Total accepted	693	(100%)	291	(100%)	984	(100%)

## Discussion

There are two important aspects of these study findings which are relevant to current global HIV programs, despite universal ART now being the global standard and the clinical trial setting being better-resourced than the public health sector. Firstly, that life-long ART uptake was slow; five sessions were required to meet the UNAIDS super-fast-track ‘Start Free’ target to reach and sustain more than 95% of HIV-infected pregnant women on life-long ART [10]. Secondly, initial life-long ART uptake was similar in settings where ART was standard and not standard for the prevention of vertical HIV transmission (64% for 1077HS and 67% for 1077BF/FF). Our study findings suggest that the the psychological and social barriers to treatment readiness among asymptomatic women were not completely overcome in the well-resourced study setting.

With the progression of WHO guidelines to recommend universal ART for all persons living with HIV, more current reports indicate successful same-day ART initiation in adult HIV clinics and maternity settings [11–13]. However, the context in which treatment is started influences retention in care, continued ART adherence and sustained virologic suppression [14, 15]. The process of initiating ART is evidently separate from overcoming barriers to remaining on ART for life.

The primary reasons given by the 65.5% who did accept the offer of universal ART mirror those reported in qualitative studies from Malawi [16], where the perception was that ART was life enhancing and led to normalization of physical appearance. These were facilitators for ART uptake among newly diagnosed HIV-infected pregnant women within the public sector. Similar sentiments were voiced in discussions published from Tanzania, Uganda and Zimbabwe [17–19], where strengthened health and the ability to care for their families was most commonly cited as a benefit of life-long ART use among mothers.

The women included in this analysis were drawn from a well-resourced clinical trial and had lived with a known HIV-infected status for 1–6 years before being offered universal ART. During that time, they had remained asymptomatic and received intense health education, counselling and support from a team of well-trained, motivated study personnel. Despite that considerable investment, one-third of the women were hesitant about initiating universal ART when first provided with the opportunity and a strong recommendation to do so. While this could be a reflection of an ineffective delivery approach, the explanations cited for needing more time identify specific barriers to treatment readiness that are less amenable to intervention at facility level; reasons given included being psychologically unprepared to make the decision and the importance of involving significant others or attending to social circumstances before making the life-long commitment.

The need for more time to consider the offer and to consult with others before initiating life-long ART was not unexpected, given the negative stigma attached to taking ART and the fear of rejection or violence from male partners [20–24]—universal challenges that prevail across diverse health settings and cultures [25–29]. Stigma and low male partner involvement are commonly cited sociocultural barriers that hinder successful ART treatment programs for women [17, 25]. Similarly, early experiences from the Malawian and Swazi ‘Option B+’ programs indicated sociocultural challenges with a same-day start of life-long ART among women learning of their HIV status in pregnancy [12, 26, 30]. However, the PROMISE study participants had been living with their HIV diagnosis for some time before ART initiation was recommended, were well engaged in care and had received significant education about their health status and psychological support to overcome potential barriers to effective treatment—all factors that were anticipated to facilitate same-day ART uptake. In the era of Treat All, the importance of addressing such barriers in the global context should remain paramount to effectively increase the treatment readiness of women who access life-long ART to promote sustained virologic suppression.

The observation that 95 women (19% of decliners) appeared to base their decision to decline universal ART on their perceived good health status and high CD4 count illustrates the difficulty faced in changing community perceptions about the role of ART in those who remain well. Counselling offered within the PROMISE clinical trial setting was performed by trained counsellors with adequate time to go over an individual woman’s questions. In public sector antenatal settings, however, concern exists over the limited staff available to provide optimal counselling services when initiating life-long ART the same day that HIV is identified [16, 17, 31]. Qualitative reports indicate that women feel rushed into making a decision to start treatment and would prefer more time to consult with partners and discuss their questions and fears [19, 26]. Due to the nature of clinical trial settings, the same does not apply to women in our study, yet our findings highlight similar hesitance among HIV-infected mothers to take up ART with the need to think more carefully about the decision cited frequently.

Apart from receiving a very high standard of care and living with their diagnosis for some years before these events, the women in this analysis differ from newly-diagnosed women routinely given life-long ART in public maternity clinics because there were few with an immediate risk of transmitting HIV to an infant (70 were breastfeeding). Thus, these PROMISE participants may have had less self-motivation to initiate HIV treatment at that particular time. Additionally, the offer was presented to them as a choice they should make individually as opposed to universal ART being the standard of care which they would actively have to opt out of if they did not feel ready. Antenatal ART initiation for HIV-infected women is a highly acceptable biomedical intervention, resulting in the dramatic decline in global pediatric HIV infections witnessed in the last 5 year period [32]. However, an evident drop in ART adherence post-delivery was reported from early public process indicators [23, 33] which could reflect low readiness to be on treatment for life or waning motivation among asymptomatic mothers to continue treatment to preserve their own health as their infants age. Indeed, in a sample of HIV-infected women interviewed in Uganda who initiated life-long ART while pregnant, the main motivation to initiate and adhere to ART was the desire to have an HIV-free baby [25, 34].

### Limitations

Although we used standardized educational materials and processes to provide the initial information about the findings of the START study for our study participants, the requirement to respond rapidly to these findings prevented the study team from controlling the approach to its delivery. Also, subsequent counselling sessions were not standardized. It is probable that study sites repeated the processes established for the initial session, however we accept that this failure to control how the information was delivered may be the reason for differential uptake across sites. Additionally, the focus of this response was to meet the ethical obligation to provide study participants with new information that could influence their decision to remain on-study, then supporting them to sort through the implications for their personal situation and communicate their choice. For this reason, the sessions were not audio-recorded. More qualitative data would provide a deeper understanding of the decision-making process.

## Conclusion

Despite removing structural and logistic challenges that hinder universal ART access in the public sector and providing intense ART education and HIV monitoring, one-third of these HIV-infected PROMISE participants did not feel ready to start universal ART for their own health. The study setting in which these events took place was better resourced than public health ART programs. Nevertheless, psychological and social challenges remained, and this study draws attention to their importance in determining treatment readiness.
